# Exogenous proline induces regulation in 2-acetyl-1-pyrroline (2-AP) biosynthesis and quality characters in fragrant rice (*Oryza sativa* L*.*)

**DOI:** 10.1038/s41598-020-70984-1

**Published:** 2020-08-18

**Authors:** Haowen Luo, Tantan Zhang, Axiang Zheng, Longxin He, Rifang Lai, Jinhai Liu, Pipeng Xing, Xiangru Tang

**Affiliations:** 1 State Key Laboratory for Conservation and Utilization of Subtropical Agro-Bioresources, South China Agricultural University, Guangzhou, 510642 People’s Republic of China; 2 Scientific Observing and Experimental Station of Crop Cultivation in South China, Ministry of Agriculture, Guangzhou, 510642 People’s Republic of China

**Keywords:** Biochemistry, Enzyme mechanisms, Enzymes, Proteins, RNA

## Abstract

Proline is one of the precursors of the biosynthesis of 2-acetyl-1-pyrroline (2-AP) which is the key and characteristic volatile component of fragrant rice aroma. In order to study the effects of exogenous proline on 2-AP biosynthesis and other grain quality attributes in fragrant rice, two *indica* fragrant rice cultivars, “*Meixiangzhan-2*” and “*Xiangyaxiangzhan*”, and one *japonica* fragrant rice, “*Yunjingyou*”, were used in present study. At initial heading stage, proline solutions at 0 (CK), 0.10 (Pro1), 0.20 (Pro2) and 0.50 (Pro3) g L^-1^ were applied as foliar spray solution to fragrant rice plants. Compared with CK, Pro1, Pro2 and Pro3 treatments significantly increased the grain 2-AP content. The significant up-regulation effects due to proline treatments were observed in the contents of proline, △1-pyrrolidine-5-carboxylic acid (P5C) and △1-pyrroline which involved in 2-AP formation. Exogenous proline application also significantly decreased the grain γ-aminobutyric acid (GABA) content. Furthermore, proline treatments enhanced the activity of proline dehydrogenase (ProDH) as well as transcript level of gene *PRODH*. On the other hand, the transcript level of gene *BADH2* and activity of betaine aldehyde dehydrogenase (BADH) decreased under proline treatments. Proline treatments (Pro2 and Pro3) also increased the grain protein content by 3.57–6.51%. Moreover, 32.03–34.25% lower chalky rice rate and 30.80–48.88% lower chalkiness were recorded in proline treatments (Pro2 and Pro3) for both *Meixiangzhan* and *Xiangyaxiangzhan* whilst for *Yunjingyou*, foliar application of proline had no significant effect on chalky rice rate and chalkiness. There was no remarkable difference observed in grain milled quality (brown rice rate, milled rice rate and head rice rate) and amylose content between CK and proline treatments. In conclusion, exogenous proline enhanced the 2-AP biosynthesis and promoted some grain quality characters of fragrant rice.

## Introduction

Fragrant rice is famous for possessing a characteristic aroma and also fetches a high price in the international market because of the good grain quality^1,2^. In the past two decades, many studies have conducted to investigate the compound of the aroma of fragrant rice. For example, the study of Widaja et al*.*^3^ showed that the number of volatile compounds detected in the aroma exceeds 300 in both fragrant and non-fragrant rice varieties. Hashemi et al*.*^4^ demonstrated there were more than 100 volatile compounds have been detected in the aroma of fragrant rice varieties. In recent years, with the development of many researches, it is established that 2-acetyl-1-pyrroline (2-AP) is the key compound in fragrant rice aroma^1,5,6^.

The process of 2-AP biosynthesis in fragrant rice is very complicated which involved many biochemical reactions while numerous studies have been conducted to understand the mechanism of 2-AP biosynthesis. An early study has evidenced that the expression of gene BADH2 which related to the betaine aldehyde dehydrogenase (BADH) activity would inhibited the 2-AP production in fragrant rice varieties^7^. The study of Mo et al.^8^ revealed a positive and significant correlation between grain 2-AP concentration and grain γ-aminobutyric acid (GABA) content. The investigation of Yoshihashi et al.^9^ revealed that the nitrogen in the 2-AP comes from proline in fragrant rice and demonstrated that the proline, ornithine and glutamic acid are the potential precursors of 2-AP. Moreover, Bao et al.^10^ and Li et al.^11^ demonstrated that proline is converted to 2-AP in three steps: First, proline is converted into △1-pyrrolidine-5-carboxylic acid (P5C) catalyzed by proline dehydrogenase (ProDH); Then, P5C is converted into △1-pyrroline which is the limiting substrate in 2-AP biosynthesis^12^; Finally, the △1-pyrroline is converted into 2-AP in fragrant rice by non-enzymatic or enzymatic reaction. Therefore, proline has an important role to play in 2-AP biosynthesis in fragrant rice.

As one of the non-essential amino acids in the human body, proline is not only one of the components of plant protein, but also one of the osmotic regulators in plant cytoplasm^13^. Previous studies revealed that proline plays important roles in stabilizing the structure of biomacromolecules, reducing the acidity of cells, detoxifying ammonia and regulating the redox potential of cells^14,15^. However, the effect of exogenous proline on fragrant rice performances especially 2-AP biosynthesis were rarely reported.

Thus, present study was conducted with the hypothesis that foliar application of proline could enhanced the 2-AP formation in fragrant rice and the objective to study the effects of exogenous proline on 2-AP biosynthesis on the physiological and molecular level.

## Results

### 2-AP content

**Figure 1:**
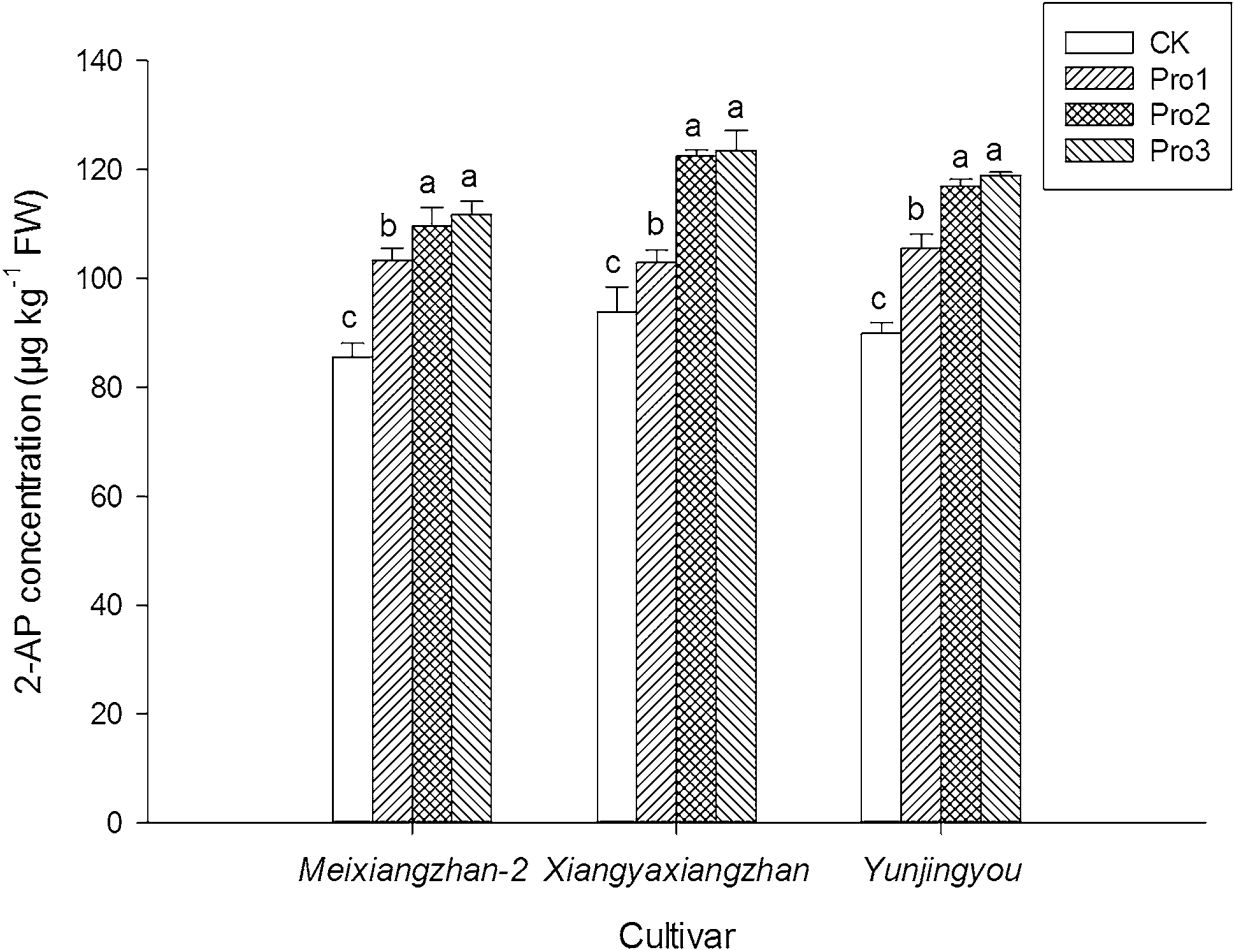
The effect of foliage dressing with proline on grain 2-AP content of fragrant rice. Data are means and standard deviation of three replications and the columns showing different letters mean the results are statistically different.

Foliar application of proline at initial heading stage significantly increased the grain 2-AP content for three fragrant rice cultivars (Fig. 1). For *Meixiangzhan-2*, compared with CK, Pro1, Pro2 and Pro3 significantly increased the grain 2-AP concentration by 20.63%, 23.24% and 23.94%. For *Xiangyaxiangzhan*, 9.65%, 27.75% and 24.18% higher 2-AP contents were recorded in Pro1, Pro2 and Pro3 than CK, respectively. For *Yunjingyou*, compared with CK, Pro1, Pro2 and Pro3 significantly increased the grain 2-AP concentration by 17.36%, 25.58% and 24.79%.

### Proline, P5C, GABA and △1-pyrroline contents

**Figure 2:**
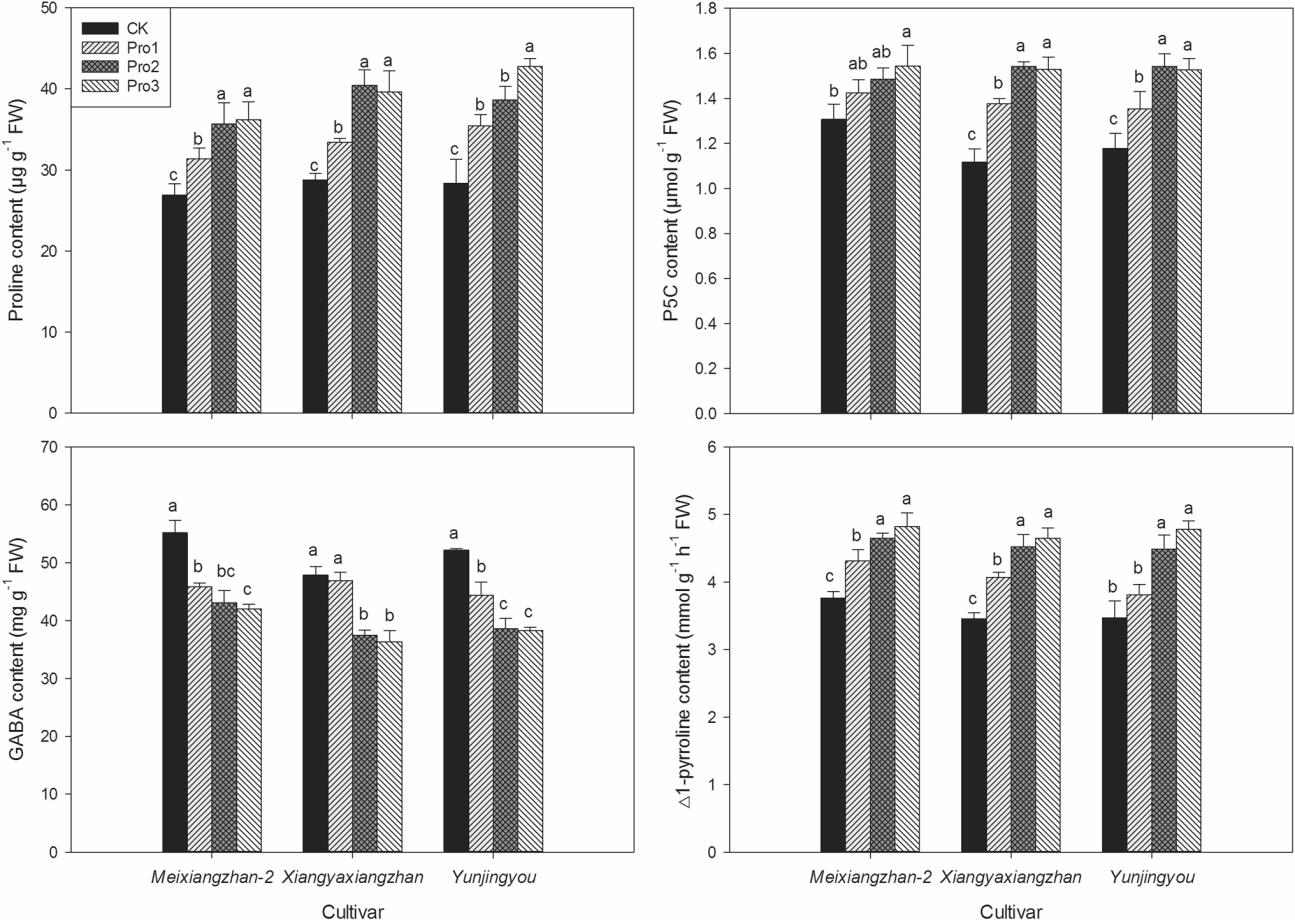
The effects of foliage dressing with proline on grain P5C, proline, GABA and △1-pyrroline contents of fragrant rice. Data are means and standard deviation of three replications and the columns showing different letters mean the results are statistically different.

As shown in Fig. 2, foliar application of proline had impacts on grain contents of proline, P5C, GABA and △1-pyrroline in fragrant rice. For proline, compared with CK, all proline treatments significantly increased the grain proline content (except Pro1 treatment in *Meixiangzhan-2*) and the highest proline contents were recorded in both Pro2 and Pro3 treatments for all fragrant rice cultivars. For P5C, higher grain P5C contents were recorded in Pro2 and Pro3 treatments than CK for three cultivars whilst there was no remarkable difference between CK and Pro1 for *Meixiangzhan-2* and *Yunjingyou*. For △1-pyrroline, higher grain △1-pyrroline concentrations were recorded in Pro1, Pro2 and Pro3 treatments than CK (except Pro1 for *Yunjingyou*) while the highest △1-pyrroline concentrations were recorded in both Pro2 and Pro3 treatments. For grain GABA content, exogenous application of proline significantly decreased the grain GABA concentration and the lowest or equally lowest contents were recorded in both Pro2 and Pro3 treatment.

### Activities of ProDH, BADH, △1-pyrroline-5-carboxylic acid synthetase (P5CS) and ornithine aminotransferase (OAT)

**Figure 3:**
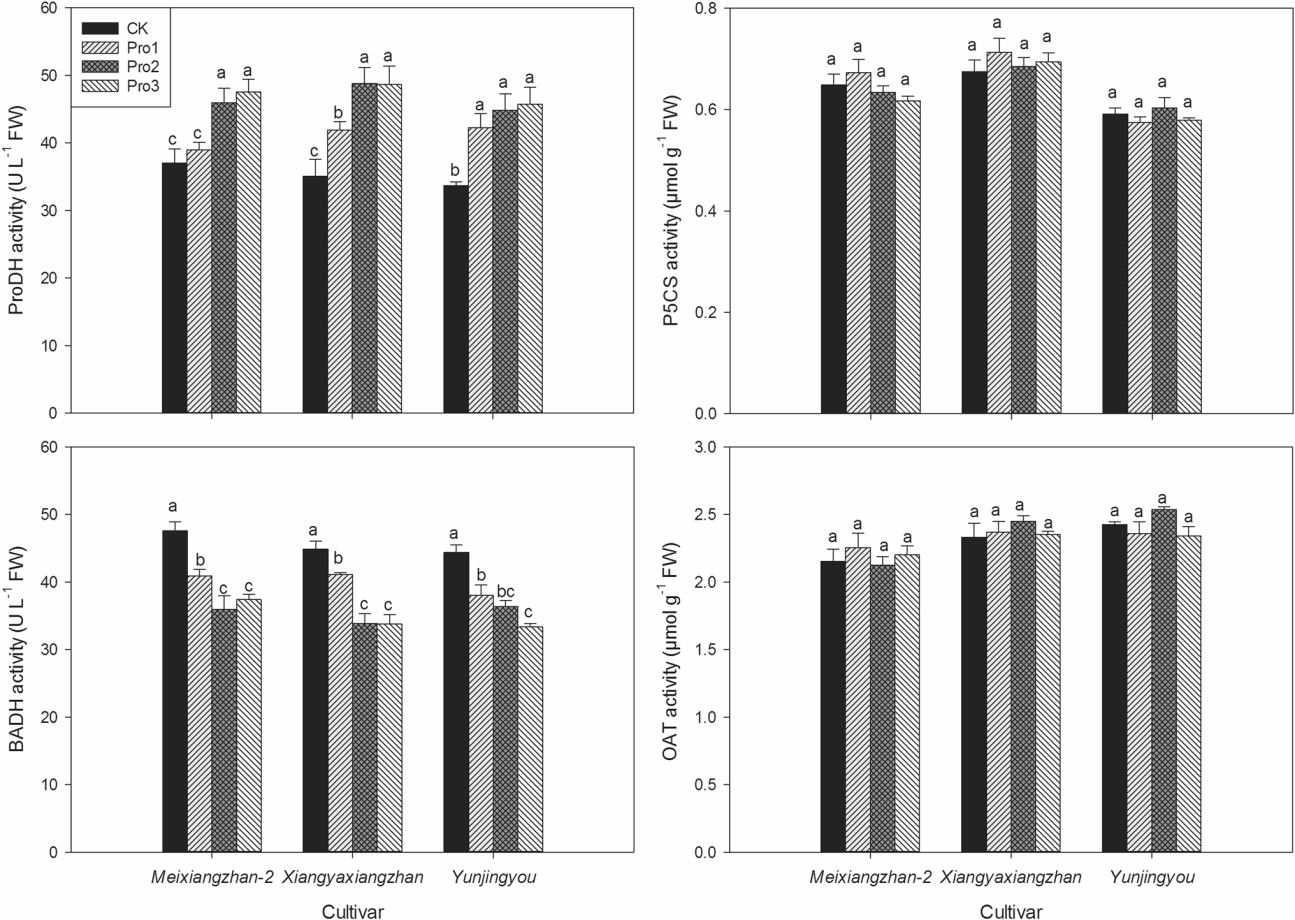
The effects of foliage dressing with proline on grain ProDH, BADH, OAT and P5CS activities of fragrant rice. Data are means and standard deviation of three replications and the columns showing different letters mean the results are statistically different.

As shown in Fig. 3, foliar application of proline at initial heading stage significantly affected the activity of ProDH. For *Meixiangzhan-2*, there was no remarkable difference between CK and Pro1 treatment whilst 24.11% and 28.57% higher ProDH activities were recorded in Pro2 and Pro3 treatments than CK and for *Xiangyaxiangzhan*, compared with CK, Pro1, Pro2 and Pro3 treatments significantly improved the activity of ProDH by 19.45%, 39.10% and 38.62%, respectively; for *Yunjingyou*, the ProDH activities under Pro1, Pro2 and Pro3 treatments were also significantly higher than CK while there was no remarkable difference among Pro1, Pro2 and Pro3 treatments. Exogenous proline application also significantly down-regulated the activity of BADH and the lowest or equally lowest activities were recorded in both Pro2 and Pro3 treatments. On the other hand, there was no remarkable differences among all treatments in P5CS activity and similar trend was also observed in OAT activity.

### Expression of genes related to 2-AP biosynthesis

**Figure 4:**
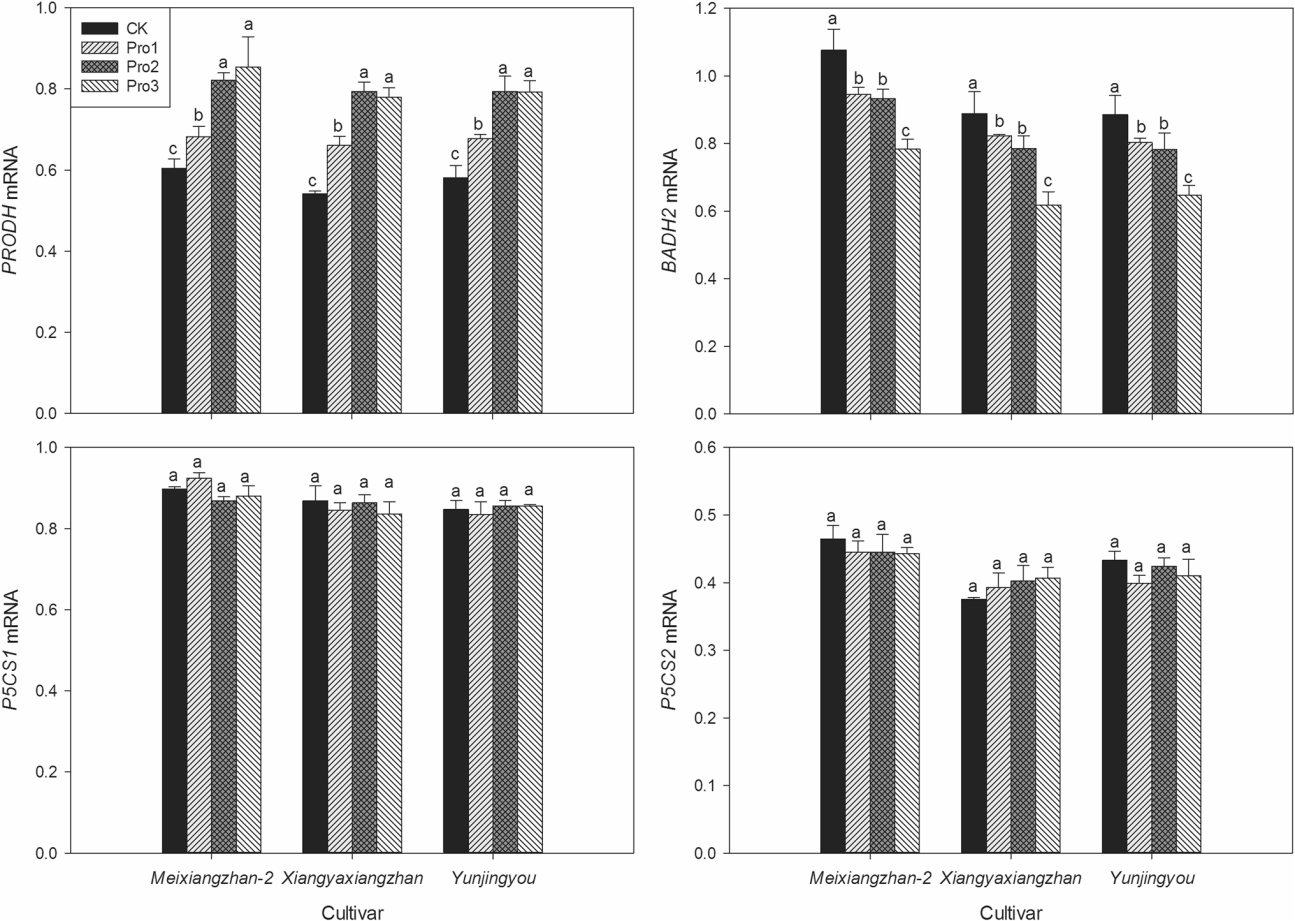
The effects of foliage dressing with proline on transcript levels of gene *PRODH*, *BADH2*, *P5CS1* and *P5CS2* of fragrant rice. Data are means and standard deviation of three replications and the columns showing different letters mean the results are statistically different.

As depicted in Real-time PCR analyses (Fig. 4), the levels of *PRODH* transcript were higher in foliar application of proline treatments. For *Meixiangzhan-2*, compared with CK, Pro1, Pro2 and Pro3 treatments significantly increased the *PRODH* transcript by 12.83%, 35.99% and 41.35%; for *Xiangyaxiangzhan*, 22.11%, 46.35% and 44.05% higher *PRODH* transcript levels were recorded in Pro1, Pro2 and Pro3 than CK, respectively; for *Yunjingyou*, Pro1, Pro2 and Pro3 significantly increased the transcript level of *PRODH* by 16.69%, 31.48% and 26.65% compared with CK, respectively. Moreover, transcript level of gene *BADH2* reduced due to exogenous proline application while the lowest or equally lowest levels were recorded in both Pro2 and Pro3 treatments. However, the transcript level of gene *P5CS1* and *P5CS2* remained not significantly different under all treatments.

### Grain yield and other quality attributes

As shown in Table 1, exogenous proline had some impacts on some grain quality attributes. Compared with CK, Pro2 and Pro3 treatments significantly increased grain protein content by 3.57–6.51% and 4.39–5.58%, respectively. Lower chalky rice rate and chalkiness were also observed in Pro2 and Pro3 than CK (except for *Yunjingyou*). However, there was no significant difference among all treatments in grain yield and compared with CK, proline treatments (Pro1, Pro2 and Pro3) had no remarkable influence on brown rice rate, milled rice rate and head rice rate as well as amylose content.

**Table 1:** The effects of foliage dressing with proline on grain yield, brown rice rate, milled rice rate, head rice rate, protein content, amylose content, chalky rice rate and chalkiness.

Cultivar	Treatment	Grain yield (t ha^-1^)	Brown rice rate (%)	Milled rice rate (%)	Head rice rate (%)	Protein (%)	Amylose (%)	Chalky rice rate (%)	Chalkiness (%)
*Meixiangzhan-2*	CK	6.23a	77.63a	64.34a	54.94a	7.17b	20.17a	16.29a	5.03a
	Pro1	6.23a	76.47a	65.24a	54.75a	7.10b	20.07a	16.71a	5.19a
	Pro2	6.20a	76.65a	65.74a	55.33a	7.63a	20.00a	10.93b	3.25b
	Pro3	6.37a	77.44a	64.89a	55.13a	7.57a	20.13a	10.79b	2.57b
*Xiangyaxiangzhan*	CK	6.30a	76.94a	65.20a	50.06a	7.47b	20.27a	16.23a	5.10a
	Pro1	6.30a	76.97a	64.60a	49.30a	7.43b	20.06a	16.29a	4.69a
	Pro2	6.40a	76.98a	65.12a	50.23a	7.73a	20.43a	10.67b	2.89b
	Pro3	6.20a	77.00a	64.35a	50.09a	7.83a	20.23a	11.03b	3.53b
*Yunjingyou*	CK	7.00a	81.09a	71.89a	62.58a	9.10b	16.90a	1.51a	0.31a
	Pro1	6.80a	81.53a	72.12a	63.60a	9.07b	17.23a	1.64a	0.30a
	Pro2	6.73a	81.51a	71.38a	63.01a	9.57a	17.23a	1.38a	0.33a
	Pro3	6.67a	81.01a	71.82a	62.44a	9.50a	17.07a	1.59a	0.29a

The means in the same column followed by different lowercase letters for the same variety differ significantly at *P* < 0.05.

## Discussion

Proline is a protein-derived amino acid with special conformational rigidity, which is essential for plant primary metabolism^13^. Early studies have revealed that proline plays multiple roles in plant physiological activities such as stress responses and protein biosynthesis^16–18^. In 2008, the report of Yoshihashi^9^ also explored that proline is one of the precursors in 2-AP production in fragrant rice cultivars. The results of present study supported the hypothesis that foliar application of proline at initial heading stage increased the grain 2-AP concentration in fragrant rice. Compared with CK, proline treatments increased the 2-AP content by 9.65–32.23%. This result agreed with the study of Yoshihashi et al*.*^9^ who demonstrated the exogenous proline greatly increased the 2-AP concentration in seedlings and callus of fragrant rice.

At the fundamental level, we observed that proline treatments increased the precursors in 2-AP biosynthesis such as proline, P5C and △1-pyrroline. Since the research of Yoshihashi et al*.*^9^, more and more studies have discovered the correlation between proline and 2-AP. For example, the study of Bao et al*.*^10^ showed that drought stress affected the 2-AP production in fragrant rice by inducing the regulation in proline biosynthesis. The investigation of Li et al*.*^19^ found a significant and positive correlation between grain 2-AP content and grain proline content under rice-duck co-culture environment. In our study, the foliar application of proline significantly increased the grain proline content and grain △1-pyrroline content in fragrant rice cultivar. Previous study revealed that △1-pyrroline is a limit factor in 2-AP production in fragrant rice^12^ and the increased △1-pyrroline concentration might be one of the reasons for the increment in 2-AP content. Moreover, present study showed that grain GABA content decreased while 2-AP content increased under the application of proline. The result was inconsistent with the research of Mo et al*.*^8^ which showed a positive correlation between GABA and 2-AP. The difference might be attributed to the experimental circumstances because the increased 2-AP concentration in study of Mo et al.^8^ was caused by stress (shading) whilst in present experiment, no any stress was made artificially and proline fitly might have effects on enhancing the stress resistance of plants^17,18^. As for the enzymes and genes related to 2-AP biosynthesis, significant differences were observed in the activities of some enzymes (ProDH and BADH) and transcript levels of some genes (*PRODH* and *BADH2*). Compared with control, proline treatments significantly enhanced the ProDH activity and gene *PRODH* expression and they were probably the further reason for increased 2-acetyl-1-pyrroline (2-AP) content under exogenous proline application. We deduced that the increased proline concentration induced the up-regulation in both ProDH activity and transcript level of *PRODH* and thus promoted the transformation from proline to P5C to △1-pyrroline and then to 2-AP^20,21^. On the other hand, as the key aroma gene in fragrant rice, the change in gene *BADH2* expression cannot be neglected and the down-regulation in *BADH2*′s transcript level and BADH activity could be other important reason for the increased grain 2-AP concentration^22,23^.

Interestingly, we observed that exogenous proline also improved some other grain quality attributes of fragrant rice. The higher grain protein content, lower chalkiness and chalky rice rates were recorded in both Pro2 and Pro3 treatments than CK. The increment in grain protein content due to exogenous proline might because the proline is one of the amino to form the plant protein and the foliar application happened to provide more proline for fragrant rice to synthesis more protein in grains^13^. On the other hand, as a trail significantly influences the appearance of rice, chalkiness has a very complicated formation which is affected by expression of many genes^24^. In our study, we observed that foliar application of proline significantly decreased chalkiness of fragrant rice cultivars, “*Meixiangzhan*” and “*Xiangyaxiangzhan*”. We deduced that there were two reasons. One was that proline is a kind of nitrogen source as described as Yoshihashi et al*.*^9^ and it might also provide nitrogen to the grain filling process while the formation of chalkiness in the endosperm is suppressed by nitrogen^25^. The other was the proline is one of the osmotic substances in plant in defending the abiotic stress^16–18^ and exogenous proline application might help to improve fragrant rice stress resistance to the potential stress from the farmland microclimate. Kong et al*.*^14^ demonstrated that environmental stress during the grain filling phase would increase chalkiness in rice grain. In order to investigate the effect of exogenous proline on rice chalkiness formation, more studies should be done to at physiological and molecular level.

In addition, the highest 2-AP contents were recorded in both Pro2 and Pro3 treatments whilst no remarkable difference was observed between Pro2 and Pro3. Therefore, 0.2 g L^−1^ might be the most suitable concentration in the proline application to increase the fragrant rice aroma considered the cost in fragrant rice production.

## Conclusion

Foliar application of proline significantly increased the grain 2-AP concentration and the related precursors including proline, P5C and △1-pyrroline in fragrant rice. At enzyme and molecular level, exogenous proline treatment significantly increased the activity of ProDH and decreased activity of BADH. The up-regulation in gene *PRODH* expression and down-regulation in gene *BADH2* expression were also observed in the proline treatments. Exogenous proline also increased grain protein and reduced chalkiness and chalky rice rate of fragrant rice.

## Methods

### Plant materials and experimental details

Two *indica* fragrant rice cultivars, “*Meixiangzhan-2*” (bred and selected by Rice Research Institute, Guangdong Academy of Agricultural Sciences) and “*Xiangyaxiangzhan*” (bred and selected by Taishan agricultural science research institute), and one *japonica* fragrant rice, “*Yunjingyou*” (bred and selected by Institute of Grain Crops, Yunnan Academy of Agricultural Sciences), were provided by College of Agriculture, South China Agricultural University and used in present study. The field experiment was conducted in Zengcheng (23°13′ N, 113°81′ E), Guangdong, China, between July and November in 2019. The experimental soil was sandy loam with of 20.12% organic matter content, 1.408% total N, 1.068% total P, and 15.767% total K. After the soaking and germination, fragrant rice seeds were sown in polyvinyl chloride trays for nursery raising. Then 15-day-old seedlings were transplanted to the field at the planting distance of 30 × 16 cm. “Special biological organic fertilizer (Ci Tian)” manufactured by Foota (Dongguan) Biotechnology Co., Ltd China comprised of N + P_2_O5 + K_2_O ≥ 26% and organic matter ≥ 25% was applied at 900 kg ha^−1^ with 60% as basal dose and 40% at tillering. All other agronomic practices i.e., pest and diseases management, and weed control were the same in all treatments by following the guidelines and standards recommended by the province^14^.

At initial heading stage, proline solutions at 0.20 (Pro1), 0.50 (Pro2) and 1.00 (Pro3) g L-1 were applied as foliar spray solution using a special Knapsack Electric sprayer (3WBD-Qianfeng Agricultural machinery, Yangjiang, Guangdong, China). The treatment which spray the distilled water was set as control (CK). The treatments were arranged in randomized complete block design (RCBD) in triplicate with net plot size of 20 m^2^. At maturity stage, fresh grains from each treatment were separated from the main stem, washed with double distilled water and stored at − 80 °C for the determination of 2-AP, proline, ProDH, P5C, △1-pyrroline and molecular analysis.

### Measurement of 2-AP content

Fresh grains about 1.00 g were homogenized in 5 mL of dichloromethane and treated for 4 h in oscillations instrument (HZS-H, China) using a frequency of 200 oscillations per minute. Grain 2-AP concentration was determinate using the synchronization distillation and extraction method (SDE) combined with GCMS-QP 2010 Plus (Shimadzu Corporation, Japan) and the grain 2-AP concentration was expressed as µg kg^-1^.

### Estimation of proline, △1-pyrroline, GABA and pyrroline-5-carboxylic acid (P5C) contents

Grain proline concentration was estimated according to the methods of SAHIN^26^ by using ninhydrin, the absorbance was read at 520 nm and expressed as ug g-1 fresh weight (FW) of leaves. The grain P5C concentration was estimated following the method of Wu^27^. The 0.9 ml reaction system contained 0.2 ml enzyme extraction, 0.5 ml trichloroacetic acid (TCA) and 0.2 ml of 2-aminobenzaldehyde. Absorbance was read at 440 nm after the reaction while grain P5C concentration was expressed as μmol g^−1^. The △1-prroline content in grains was detected according to the method described by Hill^28^. The content of △1-pyrroline in reaction mixtures containing 1,4-diaminobutane was determined immediately after 30 min in 27 °C. The estimation of grain GABA content was according to the methods described by Mo^8^ and expressed as mg g^−1^ FW.

### Determination of the ProDH, OAT, P5CS and BADH activity

ProDH activity was assayed following the methods of Li et al*.*^11^. The absorbance after reaction was read at 440 nm and the activity was calculated using a molar extinction coefficient. The estimations of activity of OAT, P5CS and BADH were according to the methods described by Bao et al*.*^10^ and expressed as μmol g^−1^ FW and U L^-1^ FW, respectively.

### Real-time quantitative RT-PCR

The total RNA in grains was extracted with HiPure Plant RNA Mini Kit (Magen, Guangzhou, China) and the cDNA was synthesize using the Hiscript II QRT SuperMix for qPCR (+ gDNA wiper) (Vazyme, Nanjing, China). Real-time quantitative RTPCR (qRT-PCR) was carried out in CFX96 real-time PCR System (Bio-Rad, Hercules, CA, USA). Each RNA sample was performed in triplicate. Primer used for qRT-PCR were listed in Table 2.

**Table 2:** Primer sequences of genes encoding enzymes involved in 2-AP synthesis in rice grains.

Gene name	Accession No	Primer sequences
*P5CS1*	AK102633	F 5′-TCTGCTCAGTGATGTGGATG-3' R 5′-CCTACACGAGATTTGTCTCC-3'
*P5CS2*	AK101230	F 5′-GAGGTTGGCATAAGCACAG-3' R 5′-CTCCCTTGTCGCCGTTC-3'
*PRODH*	AK121010	F 5′-TCATCAGACGAGCAGAGGAGAACAGG-3' R 5′-CCCAGCATTGCAGCCTTGAACC-3'
*BADH2*	AB096083	F 5′-GGTTGGTCTTCCTTCAGGTGTGC-3′ R 5′-CATCAACATCATCAAACACCACTAT-3′

### Measurement of grain yield and other grain quality attributes

At the maturity stage, the rice was harvested from three-unit sampling area (1 m^2^) in each treatment to estimate the grain yield. Then, rice huller (Jiangsu, China) was used to estimate the brown rice rate while milled rice and head rice rates were determinate by using a Jingmi testing rice grader (Zhejiang, China)^14^. The chalkiness and chalkiness degree of fragrant rice were measured with an SDE-A light box (Guangzhou, China) and the grain amylose and protein contents was measured using an Infratec-1241 grain analyzer (FOSS-TECATOR)^14^.

### Statistical analyses

The experiment data were subjected to analysis of variances (ANOVA) using Statistix 8 (Analytical software, Tallahassee, Florida, USA). The differences among means were separated by using least significant difference (LSD) test at 5% probability level. Graphical representation was conducted via Sigma Plot 14.0 (Systat Software Inc., California, USA).
